# 2-[(1-{[3-(dimethylazaniumyl)propyl]methylamino}ethylidene)azaniumyl]­nona­hydro-*closo*-deca­borate dimethyl sulfoxide disolvate

**DOI:** 10.1107/S1600536811020186

**Published:** 2011-06-18

**Authors:** Thomas D. Getman, Rudy L. Luck, Caitlin Cienkus

**Affiliations:** a Department of Chemistry, Northern Michigan University, 1401 Presque Isle Ave, Marquette, MI 49855, USA; b Department of Chemistry, Michigan Technological University, Houghton, MI, USA

## Abstract

The title compound, 2-B_10_H_9_NH=C(CH_3_)N(CH_3_)CH_2_CH_2_CH_2_N(CH_3_)_2_H·2C_2_H_6_OS or C_8_H_29_B_10_N_3_·2C_2_H_6_OS, is zwitterionic with the negative charge localized on the deca­borate cage and the positive charge on the terminal ammonium group. Two mol­ecules of dimethyl sulfoxide (DMSO) and one mol­ecule of the title compound constitute the asymmetric unit. One DMSO mol­ecule is disordered [ratio 0.739 (3):0.261 (3)]. The bonds and angles within the deca­borate cage are within the normal ranges. The amidine fragment of the ligand, which is expected to be planar, is significantly distorted from planarity as exemplified by four torsion angles [B—N—C—C = 8.4 (3), H—N—C—N = 5(2), N—C—N—C = 7.3 (3) and C—C—N—C = 14.8 (3)°] found within this portion of the mol­ecule. The crystal packing consists of head-to-tail-arranged dimers of the title mol­ecule held together by four mol­ecules of DMSO which are attached *via* strong N—H⋯O and weak C—H⋯O hydrogen bonds.

## Related literature

For related structures of 2-substituted deca­borate compounds, see: Dou *et al.* (1994 ▶); Siriwardane *et al.* (1989 ▶). For related structures of amidine-substituted polyhedral boranes, see: Froehner *et al.* (2006 ▶). For related structures involving DMSO as solvate, see: Geremia *et al.* (2000 ▶); Hulme & Tocher (2004 ▶). For structural parameters involving amidinium cations, see: Häfelinger & Kuske (1991 ▶). For related synthetic work, see: Froehner *et al.* (2006 ▶).
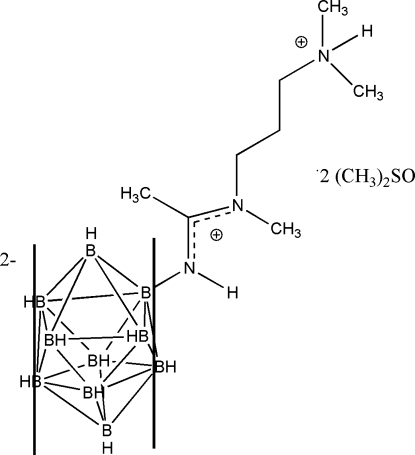


## Experimental

### 

#### Crystal data

C_8_H_29_B_10_N_3_·2C_2_H_6_OS
*M* *_r_* = 431.7
Monoclinic, 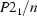

*a* = 9.503 (2) Å
*b* = 15.123 (5) Å
*c* = 18.032 (5) Å
β = 103.94 (2)°
*V* = 2515.1 (12) Å^3^
*Z* = 4
Mo *K*α radiation
μ = 0.23 mm^−1^
*T* = 564 K
0.54 × 0.42 × 0.25 mm

#### Data collection

Enraf–Nonius TurboCAD-4 diffractometer
Absorption correction: ψ scan (North *et al.*, 1968 ▶) *T*_min_ = 0.989, *T*_max_ = 1.000
5016 measured reflections
4412 independent reflections
3208 reflections with *I* > 2σ(*I*)
*R*_int_ = 0.016
3 standard reflections every 166 min  intensity decay: 1%

#### Refinement

*R*[*F* ^2^ > 2σ(*F* ^2^)] = 0.045
*wR*(*F* ^2^) = 0.135
*S* = 1.03
4412 reflections
316 parameters
3 restraints
H atoms treated by a mixture of independent and constrained refinement
Δρ_max_ = 0.32 e Å^−3^
Δρ_min_ = −0.32 e Å^−3^

### 

Data collection: *CAD-4 EXPRESS* (Enraf–Nonius, 1994 ▶); cell refinement: *CAD-4 EXPRESS*; data reduction: *XCAD4* (Harms & Wocadlo, 1995 ▶); program(s) used to solve structure: *SIR2004* (Burla *et al.*, 2005 ▶); program(s) used to refine structure: *SHELXL97* (Sheldrick, 2008 ▶); molecular graphics: *ORTEP-3 for Windows* (Farrugia, 1997 ▶) and *Mercury* (Macrae *et al.*, 2006 ▶); software used to prepare material for publication: *WinGX* (Farrugia, 1999 ▶).

## Supplementary Material

Crystal structure: contains datablock(s) global, I. DOI: 10.1107/S1600536811020186/wm2492sup1.cif

Structure factors: contains datablock(s) I. DOI: 10.1107/S1600536811020186/wm2492Isup2.hkl

Supplementary material file. DOI: 10.1107/S1600536811020186/wm2492Isup3.cdx

Additional supplementary materials:  crystallographic information; 3D view; checkCIF report

## Figures and Tables

**Table 1:** Hydrogen-bond geometry (Å, °)

*D*—H⋯*A*	*D*—H	H⋯*A*	*D*⋯*A*	*D*—H⋯*A*
N9—H91*A*⋯O211^i^	0.84 (3)	1.82 (3)	2.651 (6)	169 (3)
N9—H91*A*⋯O212^i^	0.84 (3)	1.98 (3)	2.817 (15)	172 (3)
N1—H11*D*⋯O111^ii^	0.79 (3)	2.67 (3)	3.446 (3)	168 (3)
C5—H5*C*⋯O111^ii^	0.96	2.46	3.114 (3)	125
C6—H6*B*⋯O111	0.97	2.5	3.256 (3)	135

Symmetry codes: (i) 
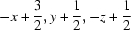
; (ii) 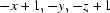.

**Fig. 1.**
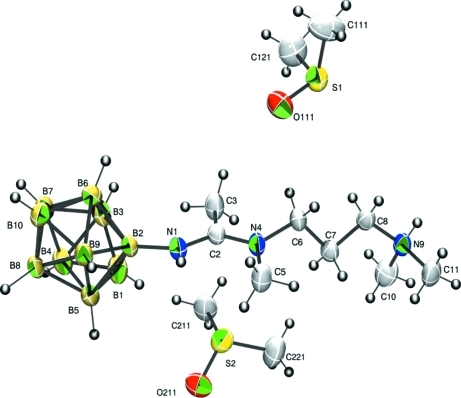
POV-RAY rendered drawing of an ORTEP-3 (Farrugia, 1997) illustration of the molecules constituting the asymmetric unit. The thermal ellipsoids are drawn at 50% probability and the H atoms are drawn as spheres of arbritary radii. Only the major orientation of the disordered DMSO molecule is shown.

**Fig. 2.**
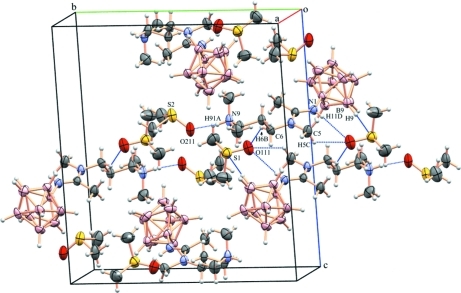
POV-RAY rendered drawing of a Mercury (Macrae et al., 2006) illustration of the H-bonded arrangement. The thermal ellipsoids are drawn at 50% probability and the H atoms are drawn as spheres of arbitrary radii. Hydrogen bonds and close contacts are shown as dashed lines and the minor orientation of the DMSO molecule is not shown. Labelled atoms are at x,y,z except for atoms S2 and O211 which were generated by symmetry code 1.5 - x,1/2 + y,1/2 - z.

## supplementary crystallographic information

### Comment

The title compound, 2-B_10_H_9_NH=C(CH_3_)N(CH_3_)CH_2_CH_2_CH_2_N(CH_3_)_2_H, (I), is zwitterionic with the negative charge localized on the decaborate cage and the positive charge on the terminal ammonium group. Two molecules of DMSO and one molecule of I constitute the asymmetric unit. One DMSO molecule is disordered over two closely situated sites with a 0.739 (3):0.261 (3) occupancy ratio. The disordered DMSO molecules display inverted conformations relative to each other, and such an arrangement was noted previously in a structure containing DMSO with a 0.95:0.05 disordered ratio (Hulme & Tocher, 2004). Fig. 1 shows the asymmetric unit, with only the major orientation of the disordered DMSO molecule drawn. Molecule I crystallizes in the form of head to tail arranged dimers held together by four molecules of DMSO which are attached *via* strong and weak hydrogen bonds as illustrated in Fig. 2. Both disordered DMSO molecules are involved with strong H-bonds involving the O atoms and a hydrogen atom bonded to atom N9 of I. As expected (Geremia *et al.*, 2000), this results in longer S—O bonds for S2—O211 = 1.509 (5) Å and S21—O212 = 1.53 (1) Å compared to the S1—O111 distance of 1.478 (2) Å where weaker interactions exist. The ordered DMSO molecule binds to two molecules of I *via* weak long range H-bonded interactions and close contacts, see Fig. 2. The H9···S1 interaction at 2.9Å is less than the sum of the van der Waals radii at 3.05Å and, given the hydridic nature of H9, is best thought of as a close contact between a hydridic hydrogen and the partially positively charged sulfur of a DMSO molecule. The "dimers" are further weakly linked to adjacent dimers *via* long range H-bonded interactions resulting in a three-dimensional linked unit.

The molecular structure of I defines a slightly distorted bicapped square antiprism with an amidine ligand coordinated to an equatorial boron atom (B2). The *exo* B—N distance, 1.541 (3) Å, in I is slightly longer than that found in [2-B_10_H_9_NCCH_3_]^-^ (II), 1.515 (5) Å, (Dou *et al.*, 1994) and [2-B_10_H_9_NCCH=CH_2_]^-^ (III), 1.523 (4) Å (Siriwardane *et al.*, 1989). The average apical-equatorial distance in I is B1—B_eq_ = 1.693 (5) Å, which compares favorably to the analogous distances of 1.682 (4) Å and 1.693 (5) Å in II and III, respectively, and B10—B_eq_ = 1.692 (11) Å, which compares favorably to the analogous distances of 1.689 (2)Å and 1.686 (5) Å in II and III, respectively. The average B—B distance between equatorial planes is 1.815 (9) Å. The average B_eq_—B_eq_ distance defined by B2, B3, B4 and B5 is 1.835 (13) Å, and that defined by B6, B7, B8 and B9 is 1.826 (5) Å. Each of these distances is similar to those found in II and III.

The amidine ligand found in I is analogous to a protonated amidinium cation, wherein the boron hydride cage substitutes for a proton on the imino nitrogen. Amidines contain two different nitrogen atoms: a formally single-bonded amide like amino nitrogen (N_am_) and a formally double-bonded imino nitrogen (N_im_). The *sp*^2^ characters of the atoms N1, C2, and N4 are supported by the sum of the bond angles about each of these atoms of 360°, 360.0° and 359.6°, respectively. The CN_im_ and CN_am_ bond lengths C2—N1, 1.318 (3) Å, and C2—N4, 1.335 (3) Å, respectively, as well as the difference between these bonds 0.016 Å, found in I are consistent with CN_im_, CN_am_ bonds, and the difference(CN_am_—CN_im_) found within other reported amidine substituted polyhedral borane molecules (Froehner *et al.*, 2006) as well as amidinium cations (Häfelinger & Kuske, 1991). The steric congestion within the amidine portion of I is illustrated by the nonplanarity of this portion of the molecule as exemplified by the following torsion angles: B2—N1—C2—C3 8.4 (3)°, H11D—N1—C2—N4 5(2)°, N1—C2—N4—C5 7.3 (3)°, and C3—C2—N4—C6 14.8 (3)°.

### Experimental

The title compound was prepared by the reaction of [2-B_10_H_9_NCCH_3_]^-^ with *N*,*N*,*N*'-trimethyl-1,3-propanediamine (see special details section). The title compound spontaneously crystallized from DMSO solution after sitting undisturbed for a period of one week. High resolution TOF MS ES+ for ^12^C_8_^1^H_30_^14^N_3_^11^B_10_^+^: calcd, 278.3370, found, 278.3379.

### Refinement

H atoms were placed at calculated positions, with C—H = 0.97 Å (ethyl) or 0.97 Å (methyl) and refined using a riding model with *U*_iso_(H) constrained to be 1.2 *U*_eq_(C) for ethyl groups and 1.5 *U*_eq_(C) for the methyl groups. The H atoms bonded to the N atoms were constrained and refined to 0.79 (3) and 0.84 (3) Å for N1—H11D and N9—H91A respectively. One DMSO molecule is disordered over two sites with a 0.739 (3):0.261 (3) ratio.

### Crystal data

**Table:**

C_8_H_29_B_10_N_3_·2C_2_H_6_OS	*F*(000) = 928
*M**_r_* = 431.7	*D*_x_ = 1.14 Mg m^−^^3^
Monoclinic, *P*2_1_/*n*	Mo *K*α radiation, λ = 0.71069 Å
Hall symbol: -P 2yn	Cell parameters from 25 reflections
*a* = 9.503 (2) Å	θ = 10–15°
*b* = 15.123 (5) Å	µ = 0.23 mm^−^^1^
*c* = 18.032 (5) Å	*T* = 564 K
β = 103.94 (2)°	Prism, colourless
*V* = 2515.1 (12) Å^3^	0.54 × 0.42 × 0.25 mm
*Z* = 4	

### Data collection

**Table:**

Enraf–Nonius TurboCAD-4 diffractometer	3208 reflections with *I* > 2σ(*I*)
Radiation source: Enraf–Nonius FR590	*R*_int_ = 0.016
graphite	θ_max_ = 25.0°, θ_min_ = 1.8°
non–profiled ω/2θ scans	*h* = 0→11
Absorption correction: ψ scan (North *et al.*, 1968)	*k* = −1→17
*T*_min_ = 0.989, *T*_max_ = 1.000	*l* = −21→20
5016 measured reflections	3 standard reflections every 166 min
4412 independent reflections	intensity decay: 1%

### Refinement

**Table:**

Refinement on *F*^2^	Primary atom site location: structure-invariant direct methods
Least-squares matrix: full	Secondary atom site location: difference Fourier map
*R*[*F*^2^ > 2σ(*F*^2^)] = 0.045	Hydrogen site location: inferred from neighbouring sites
*wR*(*F*^2^) = 0.135	H atoms treated by a mixture of independent and constrained refinement
*S* = 1.03	*w* = 1/[σ^2^(*F*_o_^2^) + (0.069*P*)^2^ + 1.0018*P*] where *P* = (*F*_o_^2^ + 2*F*_c_^2^)/3
4412 reflections	(Δ/σ)_max_ < 0.001
316 parameters	Δρ_max_ = 0.32 e Å^−^^3^
3 restraints	Δρ_min_ = −0.32 e Å^−^^3^

### Special details

**Table:**

Experimental. Synthesis: *N*,*N*,*N*'-trimethyl-1,3-propanediamnine, 0.337 g, 2.90 mmol, was added to a solution of NEt_3_H[2-B_10_H_9_NCCH_3_], 0.760 g, 2.92 mmol, in 15 ml of acetonitrile. The light yellow solution was stirred for 30 minutes, then taken to dryness on a rotary evaporator. The resulting solid was dissolved in 10 ml of 1.0 *M* sodium hydroxide. The basic solution was extracted with pentane, to remove the generated triethylamine. The resulting aqueous solution was acidified with 1.0 *M* HCl until a precipitate formed. The white precipitate was vacuum filtered and dried overnight at 50°C under dynamic vacuum. Yield of the title compound was 0.632 g, 79%. Characterization: Hi-Res TOF MS ES^+^: calcd, 278.3370, found, 278.3379, NMR spectroscopic assignments are made using the numbering in figure 1. ^11^B NMR in p.p.m.: -0.482 (B10), -3.81 (B1), -13.66 (B2), -24.86 (B4,7,8), -28.22 (B3,5,6,9). ^1^H NMR in p.p.m.: 9.24 (s,H91A), 5.85 (s, H11D), 3.35 (t, H8A,B), 3.00 (t, H6A,B), 2.96 (s, H3A,B,*C*), 2.75 (s, H10A,B,*C*,H11A,B,*C*), 2.38 (s, H5A,B,*C*), 1.83 (q, H7A,B). Protons attached to boron not resolved. ^13^C NMR in p.p.m.: 164.35 (C2), 53.85, 47.52, 42.23, 35.83, 21.89, 15.44 (remaining aliphatic carbons). MP: 193–195°C decomposition.
Geometry. All s.u.'s (except the s.u. in the dihedral angle between two l.s. planes) are estimated using the full covariance matrix. The cell s.u.'s are taken into account individually in the estimation of s.u.'s in distances, angles and torsion angles; correlations between s.u.'s in cell parameters are only used when they are defined by crystal symmetry. An approximate (isotropic) treatment of cell s.u.'s is used for estimating s.u.'s involving l.s. planes.
Refinement. Refinement of *F*^2^ against ALL reflections. The weighted *R*-factor *wR* and goodness of fit *S* are based on *F*^2^, conventional *R*-factors *R* are based on *F*, with *F* set to zero for negative *F*^2^. The threshold expression of *F*^2^ > 2σ(*F*^2^) is used only for calculating *R*-factors(gt) *etc*. and is not relevant to the choice of reflections for refinement. *R*-factors based on *F*^2^ are statistically about twice as large as those based on *F*, and *R*- factors based on ALL data will be even larger.

### Fractional atomic coordinates and isotropic or equivalent isotropic displacement parameters (Å^2^)

**Table:**

	*x*	*y*	*z*	*U*_iso_*/*U*_eq_	Occ. (<1)
B1	0.1680 (3)	−0.0714 (2)	0.23061 (16)	0.0550 (8)	
H1	0.2248	−0.0404	0.1909	0.066*	
B2	0.2214 (3)	−0.08614 (16)	0.32607 (14)	0.0360 (5)	
B3	0.0548 (3)	−0.02616 (18)	0.28008 (16)	0.0473 (7)	
H3	0.0314	0.045	0.2737	0.057*	
B4	0.0015 (3)	−0.1179 (2)	0.21324 (16)	0.0512 (7)	
H4	−0.0646	−0.1189	0.1541	0.061*	
B5	0.1694 (3)	−0.1780 (2)	0.25976 (17)	0.0482 (7)	
H5	0.2354	−0.2262	0.2374	0.058*	
B6	0.0692 (3)	−0.07928 (17)	0.37162 (15)	0.0397 (6)	
H6	0.0771	−0.039	0.4232	0.048*	
B7	−0.0838 (3)	−0.10342 (18)	0.29181 (17)	0.0461 (7)	
H7	−0.1977	−0.0822	0.2804	0.055*	
B8	−0.0033 (3)	−0.21016 (17)	0.27737 (16)	0.0436 (6)	
H8	−0.0527	−0.2734	0.2546	0.052*	
B9	0.1497 (3)	−0.18587 (16)	0.35776 (15)	0.0387 (6)	
H9	0.2211	−0.2297	0.399	0.046*	
B10	−0.0246 (3)	−0.17579 (19)	0.36349 (16)	0.0442 (6)	
H10	−0.0823	−0.2071	0.4026	0.053*	
N1	0.3759 (2)	−0.06191 (13)	0.37114 (11)	0.0379 (4)	
H11D	0.422 (3)	−0.1011 (18)	0.3941 (15)	0.057*	
C2	0.4476 (2)	0.01325 (14)	0.37510 (12)	0.0349 (5)	
C3	0.3696 (3)	0.09380 (16)	0.33963 (15)	0.0509 (6)	
H3A	0.4242	0.1218	0.3078	0.076*	
H3B	0.3587	0.1341	0.379	0.076*	
H3C	0.2757	0.0775	0.3092	0.076*	
N4	0.58795 (18)	0.01902 (11)	0.41023 (11)	0.0382 (4)	
C5	0.6728 (3)	−0.05969 (16)	0.43804 (16)	0.0521 (6)	
H5A	0.7738	−0.0446	0.4523	0.078*	
H5B	0.6566	−0.1036	0.3985	0.078*	
H5C	0.6437	−0.0827	0.4817	0.078*	
C6	0.6612 (2)	0.10402 (15)	0.43152 (13)	0.0413 (5)	
H6A	0.7104	0.1023	0.4853	0.05*	
H6B	0.5886	0.1503	0.4247	0.05*	
C7	0.7710 (2)	0.12761 (14)	0.38569 (13)	0.0395 (5)	
H7A	0.852	0.0867	0.3972	0.047*	
H7B	0.7263	0.1246	0.3314	0.047*	
C8	0.8226 (2)	0.22097 (15)	0.40810 (15)	0.0442 (6)	
H8A	0.7417	0.2612	0.3911	0.053*	
H8B	0.8519	0.2244	0.4634	0.053*	
N9	0.9463 (2)	0.25199 (13)	0.37648 (12)	0.0446 (5)	
H91A	0.957 (3)	0.3051 (19)	0.3909 (16)	0.067*	
C10	0.9142 (3)	0.2499 (2)	0.29185 (16)	0.0624 (7)	
H10A	0.9909	0.2787	0.2749	0.094*	
H10B	0.8243	0.28	0.2711	0.094*	
H10C	0.9065	0.1896	0.2747	0.094*	
C11	1.0845 (3)	0.20572 (19)	0.41085 (18)	0.0605 (7)	
H11A	1.1038	0.2091	0.4655	0.091*	
H11B	1.1623	0.2333	0.3939	0.091*	
H11C	1.077	0.1448	0.3953	0.091*	
S1	0.51507 (7)	0.32140 (4)	0.52668 (4)	0.0539 (2)	
O111	0.4605 (2)	0.23024 (12)	0.51022 (13)	0.0745 (6)	
C111	0.5090 (4)	0.3423 (3)	0.62190 (17)	0.0923 (12)	
H112	0.583	0.3085	0.6558	0.138*	
H113	0.4157	0.3256	0.6291	0.138*	
H114	0.5249	0.4041	0.6328	0.138*	
C121	0.3700 (4)	0.3916 (2)	0.48091 (19)	0.0803 (10)	
H122	0.3593	0.3894	0.4266	0.12*	
H123	0.3906	0.4512	0.4985	0.12*	
H124	0.2819	0.3721	0.4928	0.12*	
S2	0.51955 (11)	−0.00035 (6)	0.14776 (7)	0.0572 (4)	0.739 (3)
O211	0.5077 (9)	−0.0774 (4)	0.0934 (3)	0.0675 (15)	0.739 (3)
C211	0.3870 (7)	0.0760 (4)	0.1009 (4)	0.0696 (17)	0.739 (3)
H212	0.2921	0.0524	0.0983	0.104*	0.739 (3)
H213	0.3982	0.0865	0.0501	0.104*	0.739 (3)
H214	0.3985	0.1306	0.1288	0.104*	0.739 (3)
C221	0.6739 (5)	0.0601 (3)	0.1376 (3)	0.0789 (15)	0.739 (3)
H222	0.7597	0.0256	0.1571	0.118*	0.739 (3)
H223	0.68	0.1144	0.1657	0.118*	0.739 (3)
H224	0.6654	0.0729	0.0846	0.118*	0.739 (3)
S21	0.5500 (3)	0.01848 (17)	0.08259 (17)	0.0560 (11)	0.261 (3)
O212	0.500 (3)	−0.0766 (10)	0.0621 (7)	0.057 (4)	0.261 (3)
C223	0.591 (2)	0.0268 (12)	0.1855 (12)	0.142 (10)	0.261 (3)
H225	0.6873	0.0058	0.2067	0.213*	0.261 (3)
H226	0.5229	−0.0083	0.2045	0.213*	0.261 (3)
H227	0.5833	0.0874	0.1999	0.213*	0.261 (3)
C224	0.389 (2)	0.0806 (13)	0.0630 (8)	0.059 (4)	0.261 (3)
H228	0.3483	0.0823	0.0088	0.089*	0.261 (3)
H229	0.4092	0.1398	0.0819	0.089*	0.261 (3)
H230	0.3205	0.0537	0.0877	0.089*	0.261 (3)

### Atomic displacement parameters (Å^2^)

**Table:**

	*U*^11^	*U*^22^	*U*^33^	*U*^12^	*U*^13^	*U*^23^
B1	0.0530 (17)	0.070 (2)	0.0389 (15)	−0.0207 (15)	0.0048 (13)	0.0014 (14)
B2	0.0328 (13)	0.0324 (13)	0.0408 (14)	−0.0062 (10)	0.0051 (10)	−0.0027 (11)
B3	0.0460 (15)	0.0344 (14)	0.0517 (16)	−0.0045 (12)	−0.0073 (13)	0.0062 (12)
B4	0.0498 (16)	0.0574 (18)	0.0398 (15)	−0.0130 (14)	−0.0023 (12)	−0.0038 (13)
B5	0.0375 (14)	0.0510 (17)	0.0570 (17)	−0.0090 (12)	0.0131 (12)	−0.0212 (14)
B6	0.0360 (13)	0.0382 (14)	0.0420 (14)	0.0033 (11)	0.0037 (11)	−0.0047 (11)
B7	0.0315 (13)	0.0424 (15)	0.0584 (17)	−0.0011 (11)	−0.0011 (12)	−0.0048 (13)
B8	0.0319 (13)	0.0352 (13)	0.0599 (17)	−0.0058 (11)	0.0036 (12)	−0.0117 (12)
B9	0.0342 (13)	0.0286 (13)	0.0510 (15)	−0.0013 (10)	0.0057 (11)	0.0006 (11)
B10	0.0329 (13)	0.0478 (16)	0.0519 (16)	−0.0039 (12)	0.0101 (11)	0.0005 (13)
N1	0.0338 (10)	0.0338 (10)	0.0433 (11)	−0.0057 (8)	0.0038 (8)	0.0024 (8)
C2	0.0345 (11)	0.0347 (12)	0.0362 (11)	−0.0052 (9)	0.0100 (9)	−0.0008 (9)
C3	0.0403 (13)	0.0402 (13)	0.0673 (16)	−0.0060 (11)	0.0030 (11)	0.0072 (12)
N4	0.0308 (10)	0.0323 (10)	0.0494 (11)	−0.0070 (8)	0.0057 (8)	−0.0013 (8)
C5	0.0371 (13)	0.0416 (13)	0.0727 (17)	−0.0021 (10)	0.0035 (12)	0.0093 (12)
C6	0.0336 (11)	0.0398 (13)	0.0492 (13)	−0.0098 (10)	0.0076 (10)	−0.0075 (10)
C7	0.0374 (12)	0.0306 (11)	0.0503 (13)	−0.0045 (9)	0.0103 (10)	−0.0019 (10)
C8	0.0361 (12)	0.0332 (12)	0.0656 (15)	−0.0026 (10)	0.0163 (11)	−0.0038 (11)
N9	0.0352 (10)	0.0281 (10)	0.0702 (14)	−0.0050 (8)	0.0120 (9)	−0.0012 (9)
C10	0.0569 (16)	0.0615 (17)	0.0705 (19)	−0.0039 (13)	0.0187 (14)	0.0140 (14)
C11	0.0324 (13)	0.0577 (16)	0.087 (2)	−0.0005 (12)	0.0047 (13)	−0.0022 (15)
S1	0.0505 (4)	0.0492 (4)	0.0594 (4)	−0.0044 (3)	0.0084 (3)	0.0005 (3)
O111	0.0801 (14)	0.0471 (11)	0.0977 (16)	−0.0085 (10)	0.0241 (12)	−0.0160 (11)
C111	0.104 (3)	0.107 (3)	0.0568 (19)	0.036 (2)	0.0018 (18)	−0.0131 (18)
C121	0.078 (2)	0.073 (2)	0.080 (2)	0.0112 (17)	−0.0002 (17)	0.0143 (17)
S2	0.0522 (6)	0.0373 (5)	0.0858 (9)	0.0033 (4)	0.0238 (5)	0.0100 (5)
O211	0.077 (3)	0.0317 (17)	0.094 (4)	0.0092 (16)	0.020 (4)	0.003 (2)
C211	0.056 (3)	0.037 (2)	0.111 (5)	0.0094 (19)	0.010 (4)	0.001 (4)
C221	0.052 (2)	0.069 (3)	0.118 (4)	−0.008 (2)	0.023 (3)	0.016 (3)
S21	0.0507 (16)	0.0411 (15)	0.076 (2)	0.0038 (11)	0.0158 (13)	−0.0055 (12)
O212	0.074 (6)	0.032 (5)	0.054 (7)	0.012 (4)	−0.003 (8)	−0.007 (5)
C223	0.138 (18)	0.089 (13)	0.144 (17)	0.039 (12)	−0.076 (15)	−0.056 (12)
C224	0.079 (9)	0.040 (7)	0.054 (8)	0.024 (6)	0.006 (8)	0.004 (7)

### Geometric parameters (Å, °)

**Table:**

B1—B2	1.688 (4)	C6—C7	1.521 (3)
B1—B4	1.690 (4)	C6—H6A	0.97
B1—B5	1.694 (4)	C6—H6B	0.97
B1—B3	1.698 (5)	C7—C8	1.517 (3)
B1—H1	1.1	C7—H7A	0.97
B2—N1	1.541 (3)	C7—H7B	0.97
B2—B9	1.804 (3)	C8—N9	1.499 (3)
B2—B5	1.822 (4)	C8—H8A	0.97
B2—B6	1.830 (4)	C8—H8B	0.97
B2—B3	1.840 (4)	N9—C10	1.483 (3)
B3—B7	1.810 (4)	N9—C11	1.486 (3)
B3—B6	1.811 (4)	N9—H91A	0.84 (3)
B3—B4	1.827 (4)	C10—H10A	0.96
B3—H3	1.1	C10—H10B	0.96
B4—B7	1.807 (4)	C10—H10C	0.96
B4—B8	1.820 (4)	C11—H11A	0.96
B4—B5	1.852 (4)	C11—H11B	0.96
B4—H4	1.1	C11—H11C	0.96
B5—B8	1.812 (4)	S1—O111	1.478 (2)
B5—B9	1.825 (4)	S1—C111	1.761 (3)
B5—H5	1.1	S1—C121	1.777 (3)
B6—B10	1.698 (4)	C111—H112	0.96
B6—B7	1.819 (4)	C111—H113	0.96
B6—B9	1.827 (4)	C111—H114	0.96
B6—H6	1.1	C121—H122	0.96
B7—B10	1.684 (4)	C121—H123	0.96
B7—B8	1.832 (4)	C121—H124	0.96
B7—H7	1.1	S2—O211	1.509 (5)
B8—B10	1.695 (4)	S2—C211	1.767 (6)
B8—B9	1.826 (4)	S2—C221	1.774 (4)
B8—H8	1.1	C211—H212	0.96
B9—B10	1.691 (3)	C211—H213	0.96
B9—H9	1.1	C211—H214	0.96
B10—H10	1.1	C221—H222	0.96
N1—C2	1.318 (3)	C221—H223	0.96
N1—H11D	0.79 (3)	C221—H224	0.96
C2—N4	1.335 (3)	S21—O212	1.530 (14)
C2—C3	1.488 (3)	S21—C224	1.760 (19)
C3—H3A	0.96	S21—C223	1.81 (2)
C3—H3B	0.96	C223—H225	0.96
C3—H3C	0.96	C223—H226	0.96
N4—C5	1.457 (3)	C223—H227	0.96
N4—C6	1.468 (3)	C224—H228	0.96
C5—H5A	0.96	C224—H229	0.96
C5—H5B	0.96	C224—H230	0.96
C5—H5C	0.96		
			
B2—B1—B4	99.9 (2)	B10—B9—B2	113.22 (19)
B2—B1—B5	65.17 (17)	B10—B9—B5	112.57 (19)
B4—B1—B5	66.37 (18)	B2—B9—B5	60.26 (14)
B2—B1—B3	65.83 (17)	B10—B9—B8	57.48 (15)
B4—B1—B3	65.28 (18)	B2—B9—B8	101.90 (17)
B5—B1—B3	100.3 (2)	B5—B9—B8	59.50 (15)
B2—B1—H1	130.2	B10—B9—B6	57.56 (15)
B4—B1—H1	129.9	B2—B9—B6	60.54 (14)
B5—B1—H1	129.7	B5—B9—B6	102.63 (18)
B3—B1—H1	129.9	B8—B9—B6	90.37 (16)
N1—B2—B1	121.4 (2)	B10—B9—H9	117.8
N1—B2—B9	114.61 (18)	B2—B9—H9	120
B1—B2—B9	112.92 (18)	B5—B9—H9	120.3
N1—B2—B5	126.94 (19)	B8—B9—H9	131.3
B1—B2—B5	57.57 (17)	B6—B9—H9	130.5
B9—B2—B5	60.45 (15)	B7—B10—B9	99.36 (19)
N1—B2—B6	120.34 (18)	B7—B10—B8	65.64 (17)
B1—B2—B6	111.95 (19)	B9—B10—B8	65.26 (16)
B9—B2—B6	60.37 (14)	B7—B10—B6	65.10 (16)
B5—B2—B6	102.65 (16)	B9—B10—B6	65.24 (15)
N1—B2—B3	136.68 (19)	B8—B10—B6	99.59 (19)
B1—B2—B3	57.34 (17)	B7—B10—H10	130.3
B9—B2—B3	101.84 (16)	B9—B10—H10	130.4
B5—B2—B3	90.69 (17)	B8—B10—H10	130.1
B6—B2—B3	59.13 (14)	B6—B10—H10	130.3
B1—B3—B7	111.8 (2)	C2—N1—B2	130.38 (19)
B1—B3—B6	112.4 (2)	C2—N1—H11D	114 (2)
B7—B3—B6	60.32 (15)	B2—N1—H11D	116 (2)
B1—B3—B4	57.13 (17)	N1—C2—N4	121.8 (2)
B7—B3—B4	59.57 (15)	N1—C2—C3	119.04 (19)
B6—B3—B4	102.27 (18)	N4—C2—C3	119.19 (19)
B1—B3—B2	56.82 (15)	C2—C3—H3A	109.5
B7—B3—B2	101.56 (17)	C2—C3—H3B	109.5
B6—B3—B2	60.16 (14)	H3A—C3—H3B	109.5
B4—B3—B2	89.69 (18)	C2—C3—H3C	109.5
B1—B3—H3	118.5	H3A—C3—H3C	109.5
B7—B3—H3	120.7	H3B—C3—H3C	109.5
B6—B3—H3	119.9	C2—N4—C5	121.11 (18)
B4—B3—H3	131.1	C2—N4—C6	122.56 (18)
B2—B3—H3	131.3	C5—N4—C6	115.92 (17)
B1—B4—B7	112.4 (2)	N4—C5—H5A	109.5
B1—B4—B8	111.5 (2)	N4—C5—H5B	109.5
B7—B4—B8	60.66 (16)	H5A—C5—H5B	109.5
B1—B4—B3	57.58 (17)	N4—C5—H5C	109.5
B7—B4—B3	59.75 (16)	H5A—C5—H5C	109.5
B8—B4—B3	102.03 (19)	H5B—C5—H5C	109.5
B1—B4—B5	56.93 (17)	N4—C6—C7	114.05 (18)
B7—B4—B5	101.81 (19)	N4—C6—H6A	108.7
B8—B4—B5	59.12 (15)	C7—C6—H6A	108.7
B3—B4—B5	90.13 (17)	N4—C6—H6B	108.7
B1—B4—H4	118.4	C7—C6—H6B	108.7
B7—B4—H4	120.2	H6A—C6—H6B	107.6
B8—B4—H4	120.6	C8—C7—C6	107.10 (18)
B3—B4—H4	130.8	C8—C7—H7A	110.3
B5—B4—H4	131.4	C6—C7—H7A	110.3
B1—B5—B8	111.7 (2)	C8—C7—H7B	110.3
B1—B5—B2	57.26 (15)	C6—C7—H7B	110.3
B8—B5—B2	101.76 (18)	H7A—C7—H7B	108.6
B1—B5—B9	111.57 (19)	N9—C8—C7	115.02 (19)
B8—B5—B9	60.28 (15)	N9—C8—H8A	108.5
B2—B5—B9	59.29 (14)	C7—C8—H8A	108.5
B1—B5—B4	56.70 (16)	N9—C8—H8B	108.5
B8—B5—B4	59.55 (15)	C7—C8—H8B	108.5
B2—B5—B4	89.49 (17)	H8A—C8—H8B	107.5
B9—B5—B4	101.10 (18)	C10—N9—C11	111.2 (2)
B1—B5—H5	118.7	C10—N9—C8	113.50 (19)
B8—B5—H5	120.4	C11—N9—C8	112.9 (2)
B2—B5—H5	131.2	C10—N9—H91A	109 (2)
B9—B5—H5	120.8	C11—N9—H91A	107 (2)
B4—B5—H5	131.6	C8—N9—H91A	103 (2)
B10—B6—B3	112.32 (19)	N9—C10—H10A	109.5
B10—B6—B7	57.07 (15)	N9—C10—H10B	109.5
B3—B6—B7	59.83 (15)	H10A—C10—H10B	109.5
B10—B6—B9	57.20 (14)	N9—C10—H10C	109.5
B3—B6—B9	102.06 (18)	H10A—C10—H10C	109.5
B7—B6—B9	89.77 (17)	H10B—C10—H10C	109.5
B10—B6—B2	111.58 (18)	N9—C11—H11A	109.5
B3—B6—B2	60.71 (15)	N9—C11—H11B	109.5
B7—B6—B2	101.60 (18)	H11A—C11—H11B	109.5
B9—B6—B2	59.10 (13)	N9—C11—H11C	109.5
B10—B6—H6	118.5	H11A—C11—H11C	109.5
B3—B6—H6	120	H11B—C11—H11C	109.5
B7—B6—H6	131.2	O111—S1—C111	105.73 (18)
B9—B6—H6	131.3	O111—S1—C121	105.57 (15)
B2—B6—H6	120.7	C111—S1—C121	98.14 (17)
B10—B7—B4	113.1 (2)	S1—C111—H112	109.5
B10—B7—B3	113.03 (19)	S1—C111—H113	109.5
B4—B7—B3	60.68 (16)	H112—C111—H113	109.5
B10—B7—B6	57.83 (15)	S1—C111—H114	109.5
B4—B7—B6	102.73 (18)	H112—C111—H114	109.5
B3—B7—B6	59.85 (14)	H113—C111—H114	109.5
B10—B7—B8	57.48 (16)	S1—C121—H122	109.5
B4—B7—B8	60.01 (16)	S1—C121—H123	109.5
B3—B7—B8	102.22 (19)	H122—C121—H123	109.5
B6—B7—B8	90.44 (16)	S1—C121—H124	109.5
B10—B7—H7	117.7	H122—C121—H124	109.5
B4—B7—H7	119.8	H123—C121—H124	109.5
B3—B7—H7	120.1	O211—S2—C211	105.1 (4)
B6—B7—H7	130.7	O211—S2—C221	105.4 (4)
B8—B7—H7	131	C211—S2—C221	97.2 (3)
B10—B8—B5	113.03 (18)	O212—S21—C224	104.5 (12)
B10—B8—B4	111.9 (2)	O212—S21—C223	106.9 (9)
B5—B8—B4	61.33 (16)	C224—S21—C223	97.4 (7)
B10—B8—B9	57.26 (14)	S21—C223—H225	109.5
B5—B8—B9	60.22 (14)	S21—C223—H226	109.5
B4—B8—B9	102.30 (17)	H225—C223—H226	109.5
B10—B8—B7	56.87 (16)	S21—C223—H227	109.5
B5—B8—B7	102.42 (18)	H225—C223—H227	109.5
B4—B8—B7	59.32 (16)	H226—C223—H227	109.5
B9—B8—B7	89.42 (16)	S21—C224—H228	109.5
B10—B8—H8	118.3	S21—C224—H229	109.5
B5—B8—H8	119.4	H228—C224—H229	109.5
B4—B8—H8	120.2	S21—C224—H230	109.5
B9—B8—H8	131.1	H228—C224—H230	109.5
B7—B8—H8	131.6	H229—C224—H230	109.5

### Hydrogen-bond geometry (Å, °)

**Table:**

*D*—H···*A*	*D*—H	H···*A*	*D*···*A*	*D*—H···*A*
N9—H91A···O211^i^	0.84 (3)	1.82 (3)	2.651 (6)	169 (3)
N9—H91A···O212^i^	0.84 (3)	1.98 (3)	2.817 (15)	172 (3)
N1—H11D···O111^ii^	0.79 (3)	2.67 (3)	3.446 (3)	168 (3)
C5—H5C···O111^ii^	0.96	2.46	3.114 (3)	125
C6—H6B···O111	0.97	2.5	3.256 (3)	135

Symmetry codes: (i) −*x*+3/2, *y*+1/2, −*z*+1/2; (ii) −*x*+1, −*y*, −*z*+1.
